# Ultrahigh Energy Storage Density in Superparaelectric‐Like Hf_0.2_Zr_0.8_O_2_ Electrostatic Supercapacitors

**DOI:** 10.1002/advs.202300792

**Published:** 2023-04-21

**Authors:** Haiyan Chen, Lei Liu, Zhongna Yan, Xi Yuan, Hang Luo, Dou Zhang

**Affiliations:** ^1^ School of Energy and Power Engineering Changsha University of Science and Technology Changsha Hunan 410114 China; ^2^ State Key Laboratory of Powder Metallurgy Central South University Changsha Hunan 410083 China; ^3^ College of Chemistry and Chemical Engineering Central South University Changsha Hunan 410083 China

**Keywords:** electrostatic supercapacitors, energy storage, Hf0.2Zr0.8O2, low‐temperature annealing, superparaelectric‐like

## Abstract

Electrostatic capacitors attract great interest in energy storage fields due to their advantages of high power‐density, fast charge/discharge speed, and great reliability. Intensive efforts have been placed on the development of high‐energy‐density of capacitors. Herein, a novel supercapacitor with Hf_0.2_Zr_0.8_O_2_/*x*Al_2_O_3_/Hf_0.2_Zr_0.8_O_2_ (HAH*x*) is designed to improve the breakdown strength (*E*_b_) through optimizing Al_2_O_3_ (AO) film thickness. Low‐temperature annealing is first proposed to enhance the polarization difference (*P*_m_ *−P*_r_) due to the formation of dispersed polar nanoregions, which is called “superparaelectric‐like” similar to previous super‐paraelectric behavior of perovskite structures. As results, both large *E*_b_ and *P*_m_ *−P*_r_ values are obtained, leading to an ultrahigh energy storage density of 87.66 J cm^−3^ with a high efficiency of 68.6%, as well as a reliable endurance of 10^7^ cycles. This work provides a feasible pathway to improve both the polarization difference and breakdown strength of HfO_2_‐based films by the combination of insulation insertion layer and low‐temperature annealing. The proposed strategy can contribute to the realization of high‐performance electrostatic supercapacitors with excellent microsystem compatibility.

## Graphical abstract

Low‐temperature annealing combined with the insertion of Al_2_O_3_ insulating layer can promote the generation of smaller grain sizes with well‐dispersed polar nanoregions. Such characteristics can promise high maximum polarization and low remnant polarization (just like superparaelectric‐like performance), which can improve the energy storage properties of Hf_0.2_Zr_0.8_O_2_/Al_2_O_3_/Hf_0.2_Zr_0.8_O_2_ multilayer films.

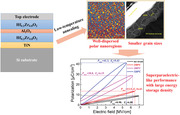

## 1 Introduction

With the increase of power requirement in modern societies, the energy storage systems are becoming crucial for energy conversion including electrochemical capacitors, Li‐ion batteries, fuel cells, and electrostatic capacitors.^[^ ^1^, ^2^, ^3^, ^4^ ^]^ Among various energy storage systems, electrostatic solid state supercapacitors of polarizable materials have received increasing interest due to their advantages of high energy density with larger power density, and are quite promising for rapid‐charge‐discharge applications.^[^ ^5^, ^6^ ^]^ Antiferroelectric dielectrics are ideal selections based on their high maximum polarization (*P*_m_) and low residual polarization (*P*_r_).^[^ ^7^, ^8^ ^]^

HfO_2_‐based anti‐ferroelectrics can achieve high energy storage densities such as Si:HfO_2_, Hf_0.3_Zr_0.7_O_2_, and Al:HfO_2_ supercapacitors,^[^ ^4^, ^7^, ^9^, ^10^ ^]^ mainly due to their larger breakdown strength (≈4–8 MV cm^−1^) and equivalent polarization value compared to that of perovskite materials.^[^ ^11^ ^]^ It is very attractive for HfO_2_‐based anti‐ferroelectrics to be used in micro‐nano electronic energy storage devices, attributed to their superior characteristics of complementary‐metal‐oxide‐semiconductor (CMOS) compatibility, mature ALD technique, excellent polarization at ultrathin thickness and environmental friendliness.^[^ ^12^, ^13^ ^]^ Park et al. reported the energy storage behaviors of Hf*_x_* Zr_1−_ *_x_* O_2_ capacitors in detail and obtained an excellent energy storage density (ESD) of 46 J cm^−3^ with an efficiency (*η*) of 51% in a 9.2 nm‐thick Hf_0.3_Zr_0.7_O_2_ antiferroelectric film.^[^ ^7^ ^]^ Kim et al. optimized the ESD and *η* to 55 J cm^−3^ and 57%, respectively, in 7 nm‐thick Hf_0.5_Zr_0.5_O_2_ films deposited at a low‐temperature of 215 °C due to the formation of more *t*‐phase.^[^ ^14^ ^]^ Pešić et al. achieved the ESD of 37 J cm^−3^ with 51% efficiency in ZrO_2_/Al_2_O_3_/ZrO_2_ antiferroelectric film capacitors, where the insulation layer was recognized to be effective for improving the breakdown strength and blocking the generation of electric tree.^[^ ^15^, ^16^ ^]^ Moreover, 3D metal‐insulation‐metal (MIM) capacitors were successfully fabricated and the total energy density could reach 930 J cm^−3^ according to the chip area.^[^ ^17^ ^]^ Although great improvements have been made so far, the critical challenge involved in Hf_1−_ *_x_* Zr*_x_* O_2_ antiferroelectric supercapacitors is the low efficiency and low ESD caused by the field‐induced phase transition from *t*‐phase to *o*‐phase especially at high electric field. Different strategies have been applied to promote the enhancement of energy storage performance, such as designation of negative capacitors and introduction of dielectric layer with much higher *ε*_r_.^[^ ^18^, ^19^ ^]^

In this work, a new concept of “superparaelectric‐like” is first applied to improve the energy storage performance in HfO_2_‐based films. Specifically, Hf_0.2_Zr_0.8_O_2_/*x*Al_2_O_3_/Hf_0.2_Zr_0.8_O_2_ (HAH*x*) multilayer structure is designed via modulating the thickness of Al_2_O_3_ (AO) insulation layers. Hf_0.2_Zr_0.8_O_2_ (H2Z8) is chosen as the antiferroelectric layer due to its excellent polarization and relatively large permittivity.^[^ ^20^ ^]^ Al_2_O_3_ has been reported that it can be effectively inhibit the development of electric tree due to its low dielectric constant (*ε_r_* ≈ 9) and large band gap, implying its high‐insulation characteristics.^[^ ^16^, ^21^ ^]^ Another uniqueness in this work is that low‐temperature annealing is proposed to decrease the grain size with smaller polar nanoregions, similar to previously reported perovskite superparaelectrics such as (Ba_0.95_, Sr_0.05_)(Zr_0.2_,Ti_0.8_)O_3_^[^ ^22^ ^]^ and Sm‐BFBT films.^[^ ^23^ ^]^ It is found that the insertion of high‐insulation Al_2_O_3_ layer can bring out more interfaces and redistribute the electric field in multilayered films. Meanwhile, low‐temperature annealing can promise fine grains, which can ensure high polarization with lower hysteresis loss. Superparaelectric‐like Hf_0.2_Zr_0.8_O_2_/10Al_2_O_3_/Hf_0.2_Zr_0.8_O_2_ film displays an ultrahigh ESD of ≈88 J cm^−3^ with a high *η* of 68.8% when the annealing temperature is 320 °C. To the best of our knowledge, it is the highest energy density ever reported in HfO_2_‐based antiferroelectric films along with relatively high efficiency. Our work can not only improve the film reliability and *ESD*, but also decrease the thermal budget, which is welcome in practical applications.

## 2 Results and Discussion

**Figure 1:**
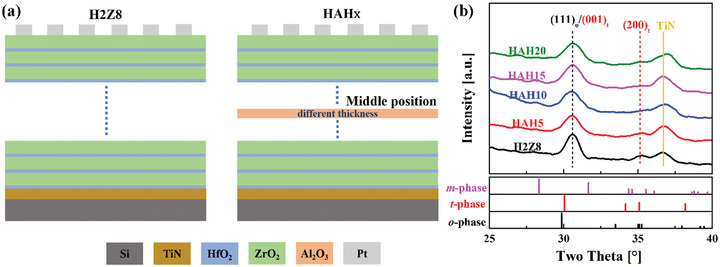
a) Schematic illustrations for H2Z8 films and HAH*x* multilayer films with different AO deposition cycles. b) GIXRD patterns of the films with different AO deposition cycles.

**Figure** **1**a shows the schematic illustrations of H2Z8 and HAH*x* films with the same total thickness of H2Z8. AO layer was inserted in the middle position of H2Z8 films with thickness range of ≈0–2.0 nm. Grazing incident X‐ray diffraction (GIXRD) patterns were employed to indicate the crystal structures of HAH*x* multilayer films and the results are shown in Figure 1b. It can be displayed that the films are polycrystalline with the coexistence of tetragonal (*t*‐) phase, orthorhombic (*o*‐) phase, and monoclinic (*m*‐) phase without obvious distinctions. The main diffraction peak is located at 30.62° and the peak is rather broad due to its small grain size. It is hard to distinguish these phases because of their similar crystal structures and close lattice constants.^[^ ^24^ ^]^ No AO peaks appear for all films, which can be explained by its amorphous characteristics at such low annealing temperature.^[^ ^25^ ^]^ The intensity of (111)_o_/(001)_t_ peak becomes weaker with the increase of AO deposition cycles. The peak in the position of 37.1° is represented as TiN electrodes, which can promise the good qualities and promote the formation of ferroelectric *o*‐phase of HAH*x* films.^[^ ^26^, ^27^ ^]^

**Figure 2:**
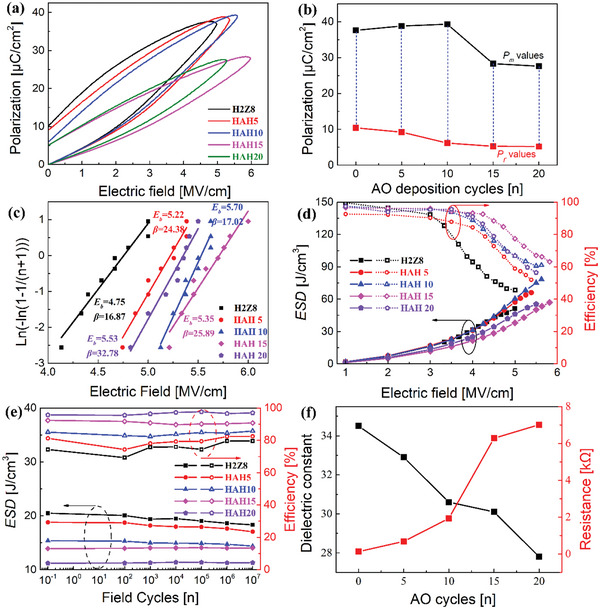
a) *P*–*E* loops and b) *P*_m_ and *P*_r_ values near their *E*_b_, c) Weibull distribution and the corresponding fitting lines, d) ESD performance and *η* under different electric field from 0 to their *E*_b_ values and e) ESD properties and *η* values under different field cycles during fatigue, f) the dielectric constant and resistance measured at 1 kHz for HAHx films with different AO deposition cycles.

*P*–*E* hysteresis loops for the H2Z8 film and HAH*x* films with different AO deposition cycles (*x* = 5, 10, 15, 20) measured near their respective *E*_b_ are shown in **Figure** **2**a. The detailed maximum polarization (*P*_m_) and remnant polarization (*P*_r_) values are recorded in Figure 2b. It is obvious that the H2Z8 film without AO insertion layer has strong antiferroelectric performance with high *P*_m_, *P*_r_ values and large hysteresis loss according to previous investigations.^[^ ^7^, ^20^ ^]^ The initial increase of *P*_m_ with increasing AO deposition cycles from 0 to 10 can be attributed to the interfacial polarization between H2Z8 and AO due to their permittivity mismatch.^[^ ^28^, ^29^ ^]^ However, the depolarization field will be induced in HAH*x* films within thicker AO layers and finally cause the reduction of *P*_m_ and *P*_r_ values.^[^ ^30^ ^]^ In HAH10 film, large polarization difference (*P*_m_−*P*_r_) of 33.15 µC cm^−2^ is achieved due to the coupling effect. For H2Z8 and HAH5 films, large *P*_m_−*P*_r_ can also be obtained which indicates their excellent energy storage performance. To investigate the puncture resistance, the statistical breakdown strength (*E*_b_) is obtained through Weibull distribution and its fitting lines (Figure 2c). *E*_b_ values increase from 4.75 MV cm^−1^ (*x* = 0) to 5.7 MV cm^−1^ (*x* = 10) and the Weibull module (*β*) is also very large, indicating the wonderful uniformity of films. The improvement of breakdown performance is ascribed to the prevention of electronic carriers by the insulate AO middle layer. Meanwhile, more interfaces can inhibit the development of electric tree and enhance the film reliability.

The corresponding ESD and *η* are derived at fields up to their *E*_b_ according to the *P*–*E* loops and the results are shown in Figure 2d, where ESD and *η* were calculated according to the following formula:

(1) $$\mathrm{ESD}\;=\int_{P_{r}}^{P_{m}}EdP\;$$

(2) $$W_{\mathrm{loss}}=\int_{0}^{P_{m}}EdP\;$$

(3) $$\eta \;=\frac{\mathrm{ESD}}{\mathrm{ESD}+W_{\mathrm{loss}}}\;\times 100\%$$

The maximum ESD of 78.32 J cm^−3^ and *η* of 61.02% are achieved in the optimized multilayer structure of the HAH10 film. However, the maximum ESD and *η* are only 51.3 J cm^−3^ and 41.64% respectively for H2Z8. The great enhancement of ESD in HAH10 is attributed to the synergistic effect of improved *E*_b_ of 5.7 MV cm^−1^ and large *P*_m_ *−P*_r_ value of 33.15 µC cm^−2^. A remarkable enhancement of *η* is critical to improve the reliability of films in practical high‐power applications, which is similar to those dielectric capacitors. The field cycling energy storage performance with the applied electric field of 3.0 MV cm^−1^ is calculated as shown in Figure 2e from the *P*–*E* loops (Figure S1, Supporting Information). With the increase of AO deposition cycles, ESD displays a decreasing trend and *η* increases much under the same applied electric field. For H2Z8 and HAH10, a wake‐up process exists with slightly increased *P*_m_ and *P*_r_ values, which causes the decrease of *η* with the field cycles increasing from the initial to 100 cycles. The wake‐up effect is previously reported to originate from the phase transition from *t*‐phase to *o*‐phase.^[^ ^31^, ^32^ ^]^ Stronger ferroelectricity in H2Z8 compared to HAH10 is also verified in the amplitude and corresponding phase transition by PFM measurements as shown in Figure S2, Supporting Information. It becomes difficult for multilayered films to have a phase transition from *t*‐phase to *o*‐phase with increasing *x*, and HAH*x* films seem more like dielectrics when *x* exceeding 10.

The dependence of frequency on dielectric constant (*ε*_r_) and resistance (*R*_s_) is displayed in Figure S3, Supporting Information, where the decrease of *ε*_r_ is attributed to a faster domain switching under high frequencies.^[^ ^33^ ^]^ Higher electronic conduction at high frequency can promote the decrease of *R*_s_. The static data are chosen at the frequency of 1 kHz as shown in Figure 2f. The decrease of dielectric constant with increasing *x* can be explained through calculation of the total equivalent capacitance in a series capacitor.

(4) $$\frac{1}{C_{\mathrm{total}}}=\frac{1}{C_{H_{2}Z_{8}}}\;+\frac{1}{C_{\mathrm{AO}}}$$

(5) $$C\;=\frac{\varepsilon_{0}\varepsilon_{r}A}{d}\;$$

(6) $$d_{\mathrm{total}}=d_{H_{2}Z_{8}}\;+d_{\mathrm{AO}}$$

where *C*, *d*, *ε* donate the capacitance, thickness, and dielectric constant, respectively. According to the above formula, *ε* of the multilayer film can be calculated by:

(7) $$\varepsilon_{r}=\frac{\varepsilon_{H_{2}Z_{8}}\varepsilon_{\mathrm{AO}}\left(d_{H_{2}Z_{8}}+d_{\mathrm{AO}}\right)}{d_{H_{2}Z_{8}}\varepsilon_{\mathrm{AO}}+d_{\mathrm{AO}}\varepsilon_{H_{2}Z_{8}}}\;$$

$\varepsilon_{H_{2}Z_{8}}$can be directly obtained to be 34.5 from Figure 2f, and $d_{H_{2}Z_{8}}$ is designed to be 15 nm. Here, *ε*_AO_ is considered to be 9 based on previous investigations.^[^ ^34^, ^35^ ^]^ Taking all these values into Equation (7), the *ε*_r_ is calculated to be 31.6, 29.31, 27.43, and 25.87 for HAH5, HAH10, HAH15, and HAH20, respectively. The minor enhancement of experimental *ε*_r_ compared to calculated values (32.9 (*x* = 5), 30.6 (*x* = 10), 30.1 (*x* = 15), and 27.8 (*x* = 20)) is possibly due to the interface effects. The accumulation of space charges at the interfaces can cause the improvement of real *ε_r_* based on Maxwell‐Wagner rule.^[^ ^36^ ^]^ Larger *E*_b_ values can also be explained through the enhancement of resistivity as shown in Figure 2e because of its stronger insulation features of AO compared to H2Z8.

**Figure 3:**
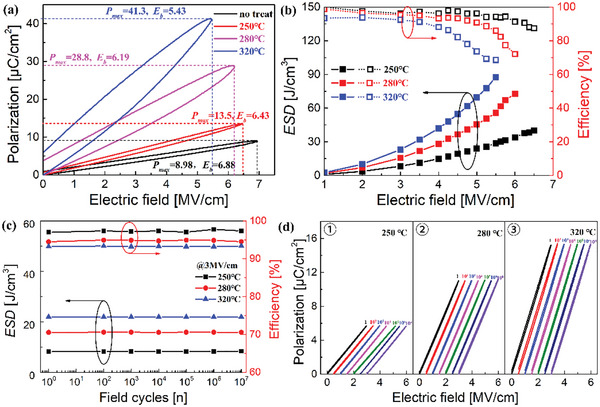
a) *P*–*E* loops of HAH10 with different annealing temperatures, b) energy storage performance of ESD and *η* as a function of electric field up to near their *E*_b_ values, c) ESD and *η* values and d) *P*–*E* hysteresis loops at 3 MV cm^−1^ under different switching cycles at various annealing temperatures of 250, 280, and 320 °C.

The formation of *o*‐phase belongs to the Martensite phase transition from *t*‐phase during cooling process for HfO_2_‐based films, where *t*‐phase is anti‐ferroelectric and *o*‐phase is ferroelectric. In order to decrease the *P*_r_ values and increase the energy storage performance, the fraction of polar *o*‐phase is decreased by controlling the annealing temperature at a quite low level based on HAH10 films, and the corresponding GIXRD result is shown in Figure S4, Supporting Information. When annealing temperature achieves at 450 °C, a little left‐shift of the (111)_o_/(001)_t_ peak from 30.64° to 30.5° represents weaker polarity. For the samples without thermal treatment and 150 °C annealing, the crystallinity is quite weak. So, we choose the annealing temperature between 250 and 350 °C, and *P*–*E* loops of HAH10 films near their *E*_b_ are characterized and shown in **Figure** **3**a. The film without annealing shows typical dielectric features with a low *P*_m_ of 8.9 µC cm^−2^. A similar dielectric characteristic for the HAH10 film with 250 °C annealing is also displayed and a slightly enhancement of *P*_m_ (13.5 µC cm^−2^) is obtained. As the *T*_anneal_ is increased to 280 and 320 °C, large *P*_m_ values of 28.8 and 41.3 µC cm^−2^ are achieved, respectively, due to their stronger crystallinity. At the same time, *P*_r_ values display a great improvement from 0.763 µC cm^−2^ (*T*_anneal_: 250 °C) to 3.9 µC cm^−2^ (*T*_anneal_: 280 °C), and finally to 6.1 µC cm^−2^ (*T*_anneal_: 320 °C), indicating the appearance of more ferroelectric *o*‐phase. This phenomenon is quite similar to those “superparaelectric” in perovskite materials with comparable *P*_m_ and much lower hysteresis loss to the corresponding ferroelectrics, and we call it “superparaelectric‐like”. The *T*_anneal_ effect on the energy storage density and efficiency of these superparaelectric‐like HAH10 films is illustrated in Figure 3b. The maximum ESD of 87.66 J cm^−3^ is obtained for the HAH10 film at *T*_anneal_ of 320 °C, which represents 71% improvement relative to H2Z8 antiferroelectric film (51.3 J cm^−3^). Meanwhile, a great enhancement of ESD value of 72.82 J cm^−3^ with *η* of 72.11% is also obtained in the film with *T*_anneal_ of 280 °C. For these superparaelectric‐like films, the degradation of *η* under fields near their *E*_b_ is caused by the combination of ferroelectric hysteresis loss and conduction loss in dielectrics. However, *η* for the film with 250 °C annealing can stay at a high level of 87.42% with relatively lower ESD of 40.08 J cm^−3^ due to its linear dielectric performance.

The reliability of energy storage performance is crucial for the practical applications of capacitors. Figure 3c shows that all these HAH10 films annealed at low temperatures maintain outstanding stability with nearly no degradations of ESD and *η* performance after 10^7^ cycles, whereas the applied electric field is 3 MV cm^−1^. The corresponding *P*–*E* loops at different field cycles are shown in Figure 3d. With the increase of annealing temperature, significant improvement of ESD values due to the enhancement of *P*_m_ values for films with stronger crystallinity. And even larger *P*_m_ value of 15.2 µC cm^−2^ (@*T*_anneal_: 320 °C) compared to 11.4 µC cm^−2^ (@*T*_anneal_: 450 °C, Figure S1a, Supporting Information) is obtained due to its larger dielectric constant of *t*‐phase than that of *o*‐phase according to the calculation of whole polarization in Equation (8).

(8) $$P\;=\varepsilon_{0}\;\varepsilon_{r}E$$

Actually, *t*‐phase is the stable phase during heating process because of its lower entropy compared to *o*‐phase and *m*‐phase. And *o*‐phase is a thermodynamically stable phase during cooling process when the kinetic energy barrier is high enough.^[^ ^37^ ^]^ Thus, the increase of both *P*_r_ values and hysteresis loss is ascribed to the formation of more *o*‐phase at higher temperatures.

**Figure 4:**
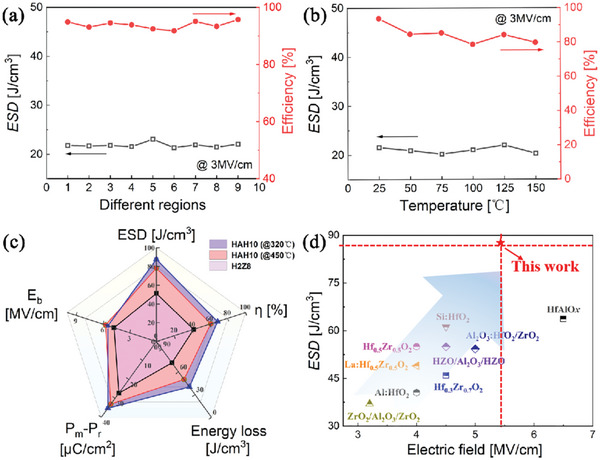
ESD properties a) at different regions and b) under different working temperatures for HAH10 at the annealing temperature of 320 °C. c) Comparison of comprehensive performance for H2Z8, HAH10 annealed @450 °C and @320 °C. d) Comparison of energy storage density and breakdown strength in our work with other previous reported HfO_2_‐based films.^[^ ^4^, ^7^, ^14^, ^17^, ^38^, ^39^, ^40^, ^41^ ^]^

In addition to the high energy storage performance at room temperature, the uniformity and temperature stability of electrostatic supercapacitors are also critical in practical applications. The obtained ESD and *η* values measured at different regions for HAH10 (@ 320 °C) show almost no changes as displayed in **Figure** **4**a and Figure S5, Supporting Information, which represents its high uniformity and consistency. Meanwhile, the temperature dependent ESD properties show little signs of degradation from 25 to 150 °C, while efficiency displays a little decreasing trend due to its larger hysteresis loss at higher temperatures from *P–E* loops as shown in Figure S6, Supporting Information. Electrical dipoles and defects like oxygen vacancies can be more active at higher temperatures, thus lead to larger hysteresis loss. The comprehensive energy storage performance of three samples (H2Z8, HAH @450 °C and HAH @320 °C) is better compared including energy storage density, efficiency, breakdown strength, energy loss, and polarization difference, as shown in Figure 4c. Significantly, ESD and *η* values are greatly enhanced through the combination effect of inserting a dielectric layer in the middle position of H2Z8 and the low‐temperature annealing. High insulation of Al_2_O_3_ layer contribute to larger *E*_b_ values in multilayered films compared to H2Z8. A greater polarization difference is observed in HAH10 (@ 320 °C) compared to HAH10 (@ 450 °C) contributes to its higher energy storage performance. Figure 4d shows the performance comparison of our work with several previous reported HfO_2_‐based films. It is clearly seen that the low‐temperature annealed HAH10 film has obvious superiority over those studies, which can provide a good pathway to obtain high‐performance energy storage properties in HfO_2_‐based films.

**Figure 5:**
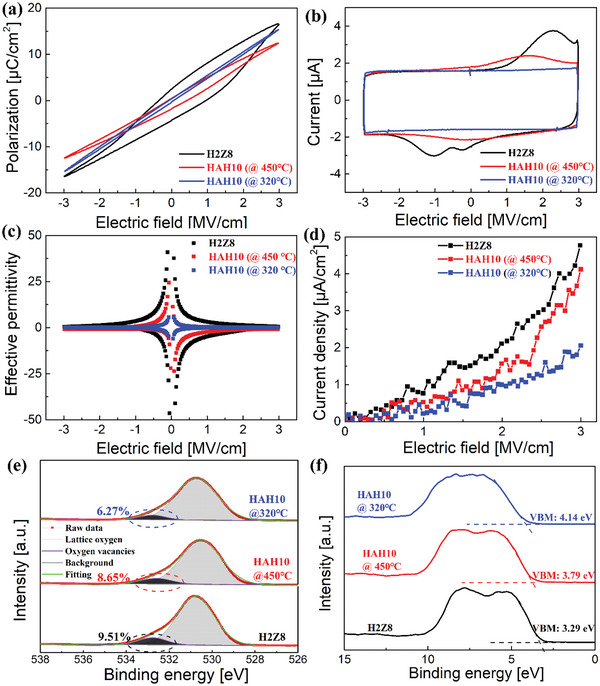
Comparison of a) P‐E loops, b) I‐E loops, c) field‐induced permittivity, d) *J*–*E* curves, e) XPS‐O 1s with high resolution and f) valence band maximum for H2Z8 and HAH10 films annealed at 450 and 320 °C.

The switching *P*–*E* and *I*–*E* loops were also characterized at the electric field of 3.0 MV cm^−1^ as shown in **Figure** **5**a,b, representing the high‐field polarization and energy storage performance. H2Z8 film shows a typical field‐induced phase transition feature with a visible *P*_r_ of 2.53 µC cm^−2^ and obvious energy loss of 17.55 J cm^−3^, along with significant double current peaks in the third quadrant. The asymmetry of *P*–*E* and *I*–*E* loops is because of the different interface conditions near top and bottom electrodes.^[^ ^42^ ^]^ A similar phenomenon is also discovered in the HAH10 film annealed at 450 °C, but the current intensity is much lower. It can be indicated that domains in the HAH10 film can be easier switched because of its smaller domain size. As the annealing temperature is decreased to 320 °C, no current peaks appear which indicates the decrease of polar regions in the film. Much decreased *P*_r_ of 0.42 µC cm^−2^ and energy loss of 1.60 J cm^−3^ can be achieved, which promotes the improvement of *η* (93.12%). This indicates that the crystal structure maintains mostly as non‐polar phase with the low‐temperature annealing for the HAH10 film, which can be better illustrated from the field‐induced effective permittivity d*P*/d*E* as show in Figure 5c. Suppressed effective permittivity of HAH10 (*@T*_anneal_: 320 °C) is due to the reduction of ferroelectric *o*‐phase in this sample. The effective permittivity varies much at low electric field below 1 MV cm^−1^, and tends to become the same at high electric field, which can promote the enhancement of energy storage and consistent to previously reported Sm‐BFBT films.^[^ ^23^ ^]^

*J*–*E* curves of these films are also measured under the electric field of 0–3 MV cm^−1^ and the results are shown in Figure 5d. The leakage current increases continuously for all films due to the dielectric conduction loss. At the electric field of 3 MV cm^−1^, the leakage current reduces from 4.77 × 10^−6^ to 2.06 × 10^−6^ A cm^−2^ for H2Z8 and HAH (@*T*_anneal_: 320 °C), respectively. The high‐field leakage current can be caused by movement of domain walls during the phase transition process. The decreased leakage is largely beneficial for the improvement of breakdown strength and energy storage performance, which can explain the enhancement of ESD and *η* in HAH10 film annealed at 320 °C. Then, the X‐ray photoelectron spectroscopy (XPS) is measured to detect the internal oxygen vacancies and valence band maximum (VBM) for these samples as shown in Figure 5e,f. In HfO_2_‐based films, oxygen vacancies are considered as key factors to induce the wake‐up effect with a large enhancement of *P*_r_ values. The fraction of oxygen vacancies in H2Z8 is the highest, which can well explain larger ferroelectric hysteresis in Figure S1, Supporting Information. Nearly no wake‐up in HAH10 (*@T*_anneal_: 320 °C) can be due to fewer oxygen vacancies in the film.^[^ ^42^ ^]^ Meanwhile, VBMs of Hf 4f for H2Z8 films and HAH10 annealed at 450 and 320 °C are 3.29, 3.79, and 4.14 eV, respectively, which are obtained from fitting the leading edge of the valence band linearly by XPS survey data within the binding energy between 0 and 15 eV. A larger VBM for HAH10 (*@T*_anneal_: 320 °C) represents its stronger electrical resistance, which is beneficial for the improvement of breakdown strength. Other XPS data of Hf, Zr, and Al are shown in Figure S7, Supporting Information, where no significant difference exists in the films.

**Figure 6:**
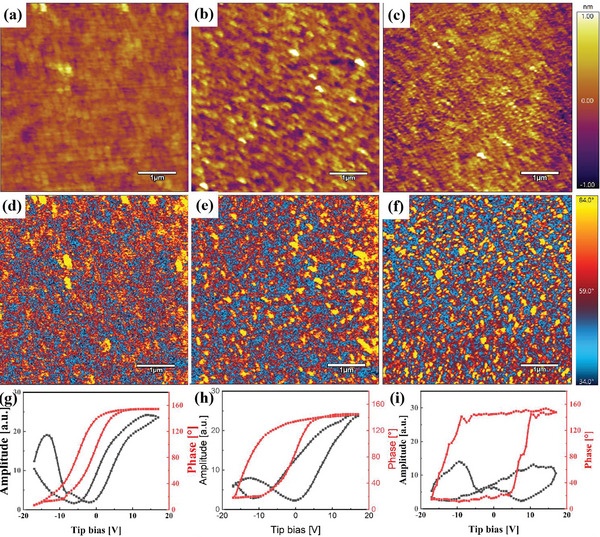
The topography (top row) and phase images (middle row) of as‐grown state, PFM hysteresis loops in amplitude and phase after applying voltage pulses of ±17 V (bottom row) for a,d,g) H2Z8, b,e,h) HAH10 (@ 450 °C) ,and c,f,i) HAH10 (@ 320 °C).

In order to further analyze the ferroelectric polarization, piezoelectric force microscopy (PFM) was performed to obtain more insights. **Figure** **6**a–f displays the topography and phase images of the as‐grown state for H2Z8, HAH10 (*@*450 °C), and HAH10 (@320 °C) films. It can be clearly seen that grain sizes are quite different from each other, which is consistent with the XRD results as shown in Figure 1b. Compared with larger strip‐like domains H2Z8, a much smaller domain size was found in superparaelectric‐like HAH10 (@320 °C) just like nano‐clusters and even nano‐domains through comparing Figure 6d with Figure 6f. The appearance of domains with smaller sizes is due to the growth of smaller crystalline grains through insertion of an ultrathin dielectric layer of Al_2_O_3_ utilizing low‐temperature annealing.^[^ ^22^ ^]^ Then, the retention behaviors of switched domains with half split pattern in all films were characterized and as shown in Figure S8, Supporting Information. The retention measurement is done at ±12 V in order to avoid breakdown of all samples with continuous voltage scanning. Two defined 2.5 × 2.5 µm^2^ regions with clear phase contrast were first polarized by a positive and negative bias of ±12 V, respectively. Then, a series of retention phase images were obtained via a grounded PFM tip by scanning a square region (5 × 5 µm^2^) of the bipolar domain pattern immediately, and after 2 and 5 min. All the films exhibit typical phase reverse after applying tip bias immediately in the PFM phase images, which comes from the electric field induced antiferroelectric‐to‐ferroelectric phase transition. The quite small phase contrast is ascribed to the fine ferroelectric domains and weak ferroelectricity of H2Z8 and HAH10 films. Followed by removing the applied electric field, the reversed domains have mostly relaxed back to the as‐grown state as shown in Figure 6d–f due to the intrinsic volatile antiferroelectricity of HfO_2_‐based films in this work. Tracking PFM images reveal that domains in H2Z8 sample relax much slower than those in HAH10 (@450 °C) and HAH10 (@320 °C). Better retention behavior in H2Z8 is ascribed to the switching of more domains due to its larger *P*_r_ value as shown in Figure 3a.

Single point hysteresis loop showing a phase loop and a typical butterfly‐like amplitude loop for all films are also displayed in Figure 6g–i. All the samples exhibit ferroelectric‐like behaviors especially with the stimulation of large voltages, such as butterfly amplitude loops, and large phase flips are observed at the minimum amplitude. However, the phase transition does not reach 180° due to the existence of non‐ferroelectric phase and other defects. The asymmetry of hysteresis loops can be originated from imprint effect.^[^ ^43^ ^]^ The superparaelectric‐like film sustaining much lower amplitude intensity is consistent with the change tendency of macroscopic *P–E* loops, which accompanies with the growth of smaller domain sizes.^[^ ^44^, ^45^ ^]^ The coercive voltage of these three samples is quite different from each other. Large coercive voltage in HAH10 (@320 °C) symbolizes that it is more difficult for films to transform into ferroelectric. The existence of well‐dispersed polar nanodomains is beneficial for the improvement of energy storage performance for superparaelectric‐like films.

**Figure 7:**
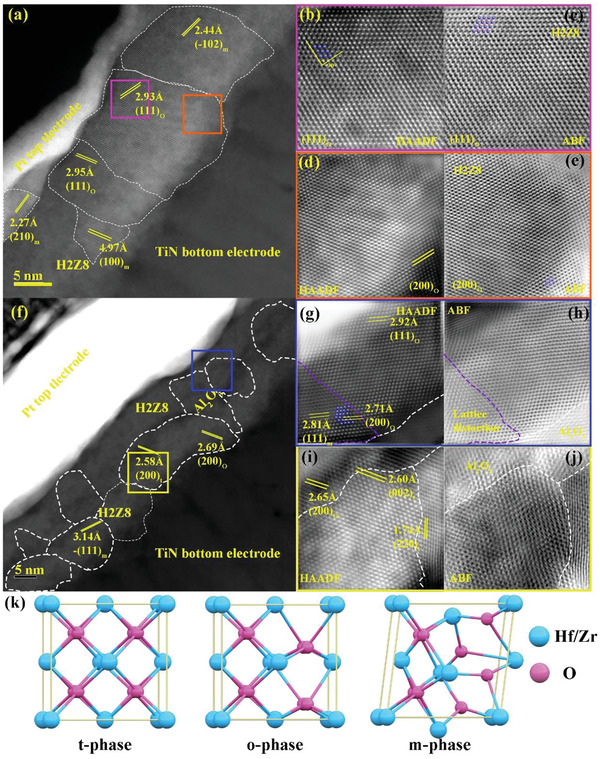
Metal–ferroelectric–metal (MFM) electrostatic supercapacitors. Cross‐sectional STEM results of a) H2Z8 and f) HAH10 (@ 320 °C), b,d) HAADF‐STEM and c,e) ABF‐STEM images near top electrode and bottom electrode, respectively, for H2Z8. g,i) HAADF‐STEM and h,j) ABF‐STEM images near top electrode and bottom electrode, respectively, for HAH10 (@320 °C). k) The crystal structures of different phases with arrangement of Hf/Zr and O atoms.

Nanograins in antiferroelectric H2Z8 and superparaelectric‐like HAH10 (@ 320 °C) films are directly characterized with atomic resolution spherical aberration‐corrected scanning transmission electron microscopy (STEM). The comparison of cross‐sectional STEM images is as shown in **Figure** **7**a,f. Clear interfaces between the uniform H2Z8 film and TiN bottom electrodes and Pt top electrodes are observed. It is visible that polycrystals with different lattice planes exist in these two films including identified (111)_o_, (200)_o_, (002)_t_ and (111)_m_ as shown in Figure 7a,c and Figure S9a–d, Supporting Information, which is consistent to the GIXRD results. Meanwhile, much smaller grain size and weaker crystallinity in HAH10 are observed compared to those in H2Z8, resulting from the combination of disruption of grain growth by the insertion of a dielectric Al_2_O_3_ layer and the low‐temperature annealing. Though the thickness of Al_2_O_3_ layer is ultrathin, the obvious contrast compared to H2Z8 indicates its atomic level deposition of ALD technic. It is generally acknowledged that *o*‐phase is the origin of ferroelectricity and can attribute to a large remnant polarization.^[^ ^20^, ^46^ ^]^ According to previous investigations, ferroelectrics can transform into linear dielectrics when the internal grain size is reduced into the single domain region for perovskite structures.^[^ ^47^, ^48^ ^]^ It is also reported that ferroelectricity can be weaker for films with smaller grain sizes for HfO_2_‐based films.^[^ ^49^ ^]^ Meanwhile, the critical grain size of HfO_2_ and ZrO_2_ is ≈4 and ≈32 nm, respectively, so the critical size of H2Z8 is calculated to be 26.4 nm according to Vegard's Law.^[^ ^50^ ^]^ Thus, larger grain size below the estimated value of 26.4 nm and larger domain size can contribute to larger *P*_r_ values, which can well explain larger ferroelectricity in H2Z8 at high fields.

In order to further elaborate the functions of A_2_O_3_ dielectric layer and low‐temperature annealing, high‐resolution high‐angle annular dark‐field (HAADF) and annular bright‐field (ABF) STEM images are displayed about the interface atomic structures in these two films as shown in Figure 7b–e and Figure 7g–j. Details of the mismatch and relationship between films and top/bottom electrodes can be clearly found. In the HAADF images, the atomic arrangement of heavy Hf/Zr atoms has no difference from each other, so the obtained ferroelectricity is originated from the displacement of the O atoms. The orientation of lattice plane is continuous for H2Z8 and the domains are large. The *t*‐phase existed in the interfacial region near TiN bottom electrode can be transformed into ferroelectric *o*‐phase under the stimulation of applied electric field.^[^ ^31^, ^32^ ^]^ At annealing temperature of 320 °C, it is quite different for grains near top electrode from those near bottom electrode. The *o*‐phase can be found near Pt top electrode due to the clamping stress, which can promise large maximum polarization. The decrease of domain size in HAH10 can also lower the remnant polarization of films. However, there exists lattice distortion in the proximity area of AO dielectric layer and lattice constant is reduced obviously, mainly because of its smaller atom radius of Al compared to Hf/Zr.^[^ ^51^ ^]^ The *t*‐phase is also formed at the interface near TiN bottom electrode. However, independent grains and dispersed domains are formed in HAH10 (@320 °C) compared to the columnar growth of grains and stripped large domains in H2Z8. Thus, the combination of AO insertion layer and low‐temperature annealing is quite beneficial for the inhibition of ferroelectricity. Meanwhile, the existence of small polar regions is advantageous to improve the superparaelectric‐like characteristics and energy density of films.

## 3 Conclusion

In this paper, an ultrahigh energy storage density of 87.66 J cm^−3^ and efficiency of 68.6% together with large breakdown strength of 5.5 MV cm^−1^ were achieved in the HAH10 supercapacitor. The excellent results are attributed to the enhanced breakdown strength through insertion of an insulation AO layer and the superparaelectric‐like properties due to the dispersed polar nanoregions at a low annealing temperature. Moreover, low‐temperature annealed films can display fatigue‐free properties after 10^7^ voltages cycling and wake‐up free properties for HAH10. Low temperature cannot provide enough driving force for the phase transition from *t*‐phase to ferroelectric *o*‐phase, which can effectively decrease the remnant polarization to a low value. Meanwhile, more *t*‐phases with higher dielectric constant can promise the high maximum polarization, which is also beneficial for the improvement of energy storage density. Based on advantages of the CMOS compatibility and easy integration, HfO_2_‐based supercapacitors are potential in power supply for future micro‐nano electronics.

## 4 Experimental Section

### Raw Materials

Ti metal target (*Φ* = 60 mm, *t* = 3 mm, 99.99%) and Pt metal target (*Φ* = 60 mm, *t* = 3 mm, 99.99%) were provided by Zhongnuo New Materials (Beijing) Technology Co. Ltd. Single‐crystal substrates of (100) Si (0.5 mm) was provided by Anhui Institute of Optics and Fine Mechanics, Chinese Academy of Sciences. Metal organic precursors of tetrakis‐ethylmethylamino‐hafnium (TEMAH), tetrakis‐ethylmethylamino‐zirconium (TEMAZ), and trimethylaluminium (TMA) were provided by Suzhou Fornano Electronic Technology Co. Ltd.

### Film Deposition

TiN bottom electrodes were deposited by a reactive sputtering method using Ti targets in nitrogen atmosphere. Hf_0.2_Zr_0.8_O_2_ films were deposited by alternately repeating two cycles of HfO_2_ and eight cycles of ZrO_2_ at the chamber temperature of 250 °C. The growth rate of HfO_2_ and ZrO_2_ was 0.091 nm per cycle, 0.084 nm per cycle, respectively according to the previous investigation by the authors.^[^ ^52^ ^]^ In this work, the final thickness of Hf_0.2_Zr_0.8_O_2_ film was all set as ≈15 nm. Al_2_O_3_ films were all deposited in the middle position of Hf_0.2_Zr_0.8_O_2_ film at the same chamber temperature of 150 °C with the film thickness of 0, 0.5, 1.0, 1.5, and 2.0 nm by tailoring the deposition cycles of 0, 5, 10, 15, and 20 cycles, respectively. Pt top electrodes were DC sputtered on the surface of these films using a circular shadow mask (*Φ* = 200 µm) at room temperature. After coating, the HAH*x* (*x* donates deposition cycles) films with different AO thickness were annealed at 450 °C for 60 s in a rapid thermal treatment. To further modulate the paraelectric and ferroelectric performance, HAH10 films were annealed under different temperatures from 250 to 320 °C for 60 s.

### Characterizations

The crystallographic structures of metal‐ferroelectric‐metal capacitors (TiN/HAH𝑥/Pt) were analyzed via GIXRD scans in a commercial D/max 2550 XRD diffractometer system (equipped with a Cu K*α* radiation source, Japan). The diffractograms were obtained under the grazing angle of 3° with the integration step of 0.2°s^−1^. Polarization‐electric field (*P*–*E*) loops and current‐electric field (*I*–*E*) loops were measured by TF Analyzer 2000 in a virtual ground mode. A triangle wave was applied to the top electrode to collect the data while the bottom electrode was connected to the ground at the frequency of 1 kHz.

The static capacitance and resistance were obtained at 1 kHz using an impedance analyzer (4294A, Agilent Technologies, USA). Element species and their bonding states were determined by XPS (ESCALAB250Xi, Thermo), where all high‐resolution spectra were calibrated through setting C 1s at 285 eV. Piezoresponse force microscopy (Asylum Research MFP‐3D Infinity) was used to detect the polar domain structures. Before PFM measurements, polarization states were realized on preselected local areas of 2.5 × 2.5 µm^2^ by applying preset positive and negative tip bias. The retention behavior was performed by scanning a region of 5 × 5 µm^2^, while the probe and the bottom electrode were both grounded. The sample for HRTEM analysis was prepared by the focused ion beam technique (FEI, Helios Nanolab G3 system), and STEM images including HAADF‐STEM and ABF‐STEM were confirmed by the JEM Grand ARM300F microscopy. The phase identification was performed by comparing the crystal plane spacing between measured value in the HRTEM images and the standard value in the PDF card.

## Conflict of Interest

The authors declare no conflict of interest.

## Supporting information

Supporting Information

## Data Availability

The data that support the findings of this study are available from the corresponding author upon reasonable request.
