# Supplementary material for: Ultrahigh Energy Storage Density in Superparaelectric‐Like Hf0.2Zr0.8O2 Electrostatic Supercapacitors
Source: Adv Sci (Weinh). 2023 Apr 21;10(18):2300792. doi: 10.1002/advs.202300792 (PMC10288225; doi:10.1002/advs.202300792)
Supplement: Supplementary file 1 — Supporting Information [file ADVS-10-2300792-s001.pdf]

ADVANCED SCIENCE
Open Access

Supporting Information

for *Adv. Sci.*, DOI 10.1002/advs.202300792

Ultrahigh Energy Storage Density in Superparaelectric-Like $Hf_{0.2}Zr_{0.8}O_2$ Electrostatic Supercapacitors

Haiyan Chen, Lei Liu, Zhongna Yan, Xi Yuan, Hang Luo* and Dou Zhang**

Supporting Information

Ultrahigh energy storage density in superparaelectric-like $Hf_{0.2}Zr_{0.8}O_2$ electrostatic supercapacitors

Haiyan Chen, Zhongna Yan, Hang Luo, Dou Zhang**

H. Y. Chen, Z. N. Yan

School of Energy and Power Engineering

Changsha University of Science and Technology, Changsha, Hunan 410114, China

H. Luo, D. Zhang

State Key Laboratory of Powder Metallurgy,

Central South University, Changsha, Hunan 410083, China

E-mail: dzhang@csu.edu.cn, hangluo@csu.edu.cn

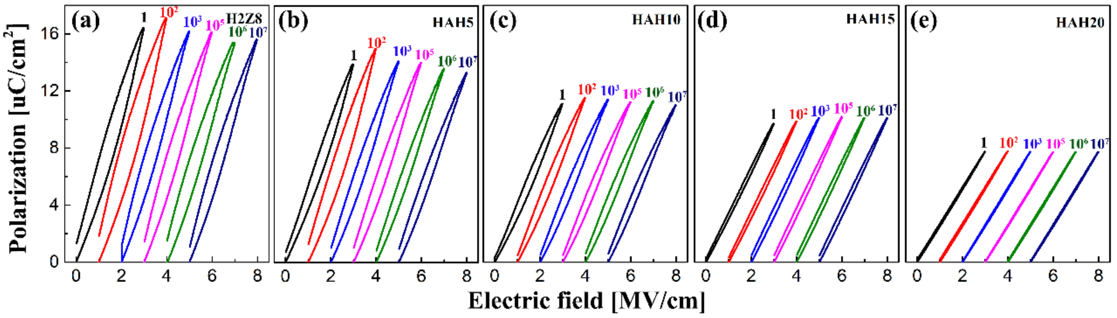

Figure S1. Fatigue performance at different field cycles for HAH*x* films.

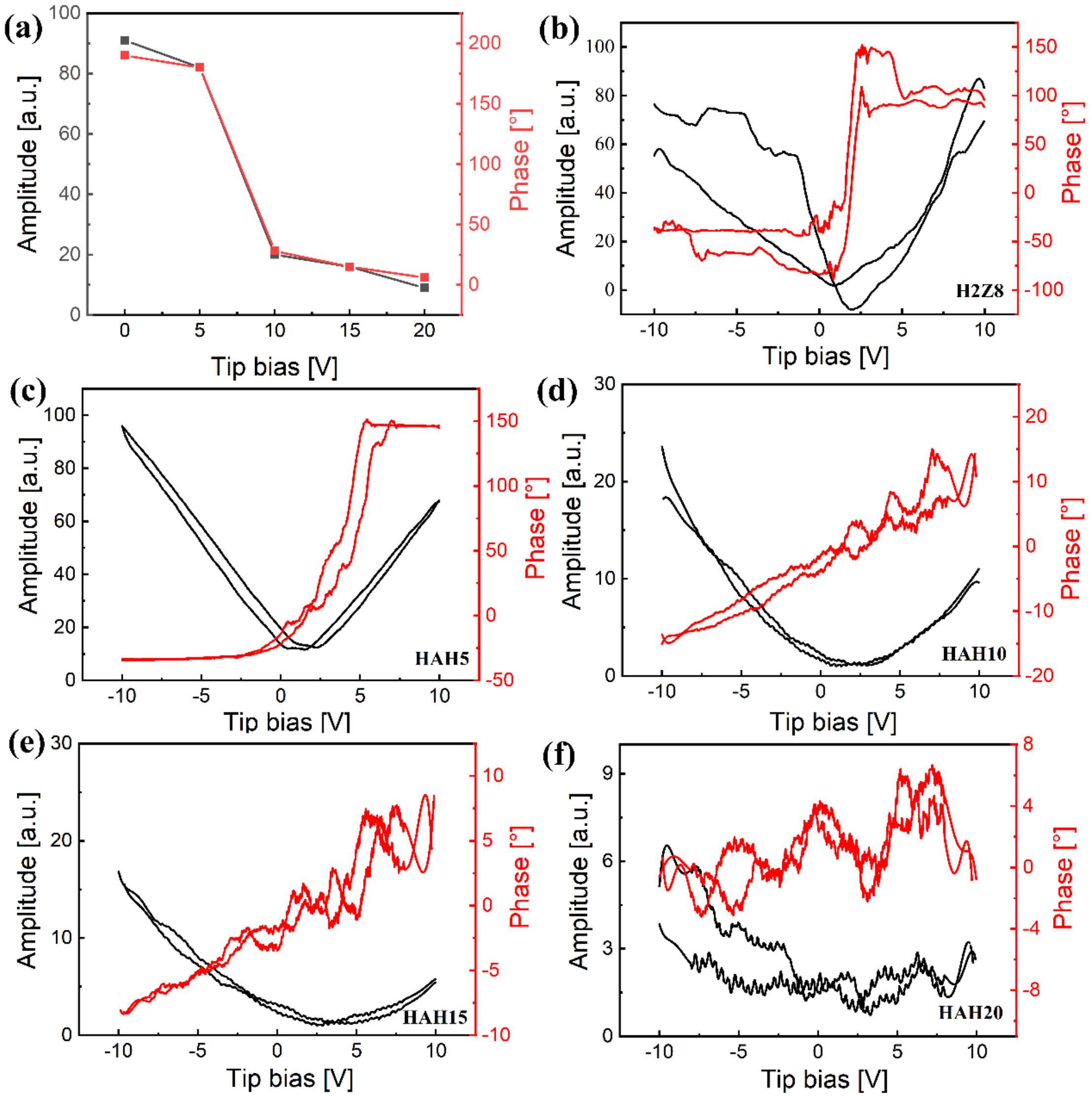

Figure S2. PFM amplitude and phase for all HAH*x* films

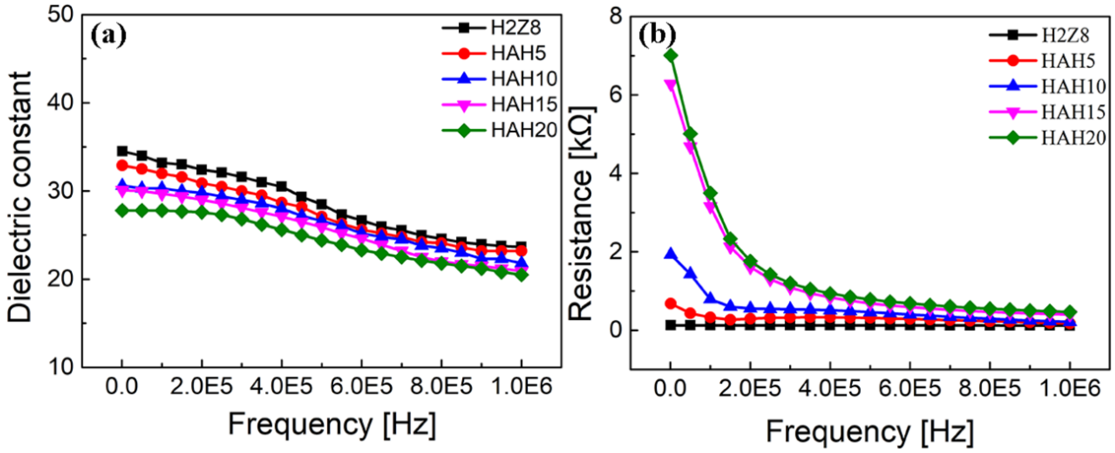

Figure S3. The dependence of frequency on
(a) dielectric constant and (b) resistance from o Hz to 10^6 Hz.

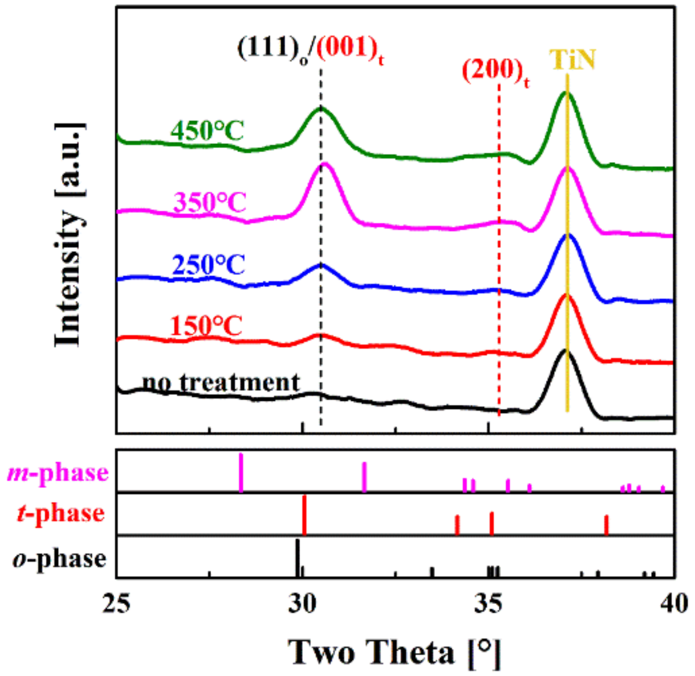

Figure S4. GIXRD results of HAH10 annealed at different temperatures.

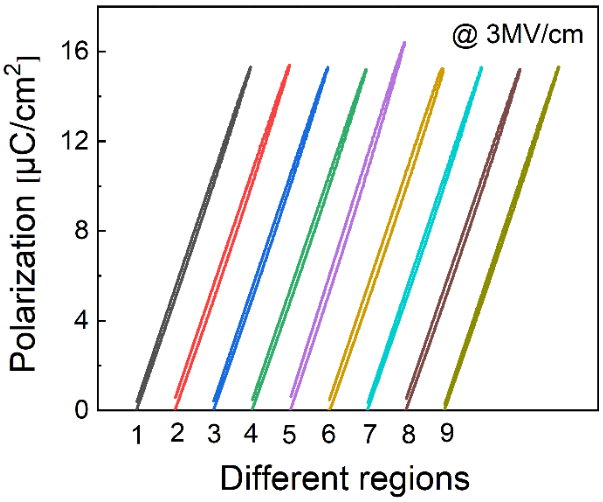

Figure S5. P-E loops measured at different regions in HAH10 (@ 320 ℃).

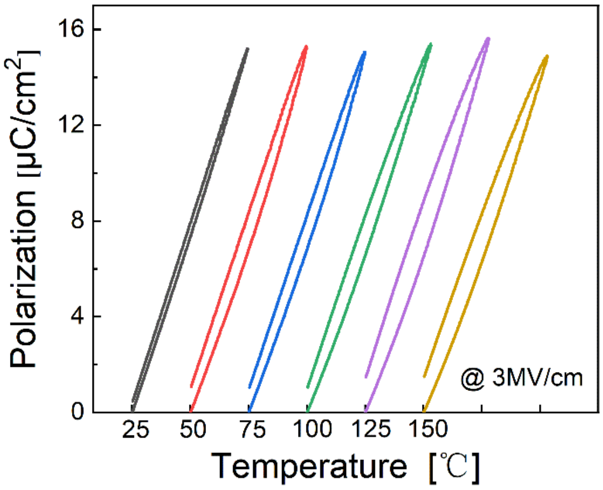

Figure S6. *P-E* loops measured at different temperatures in the range of 25 ℃~150 ℃ in HAH10 (@ 320 ℃).

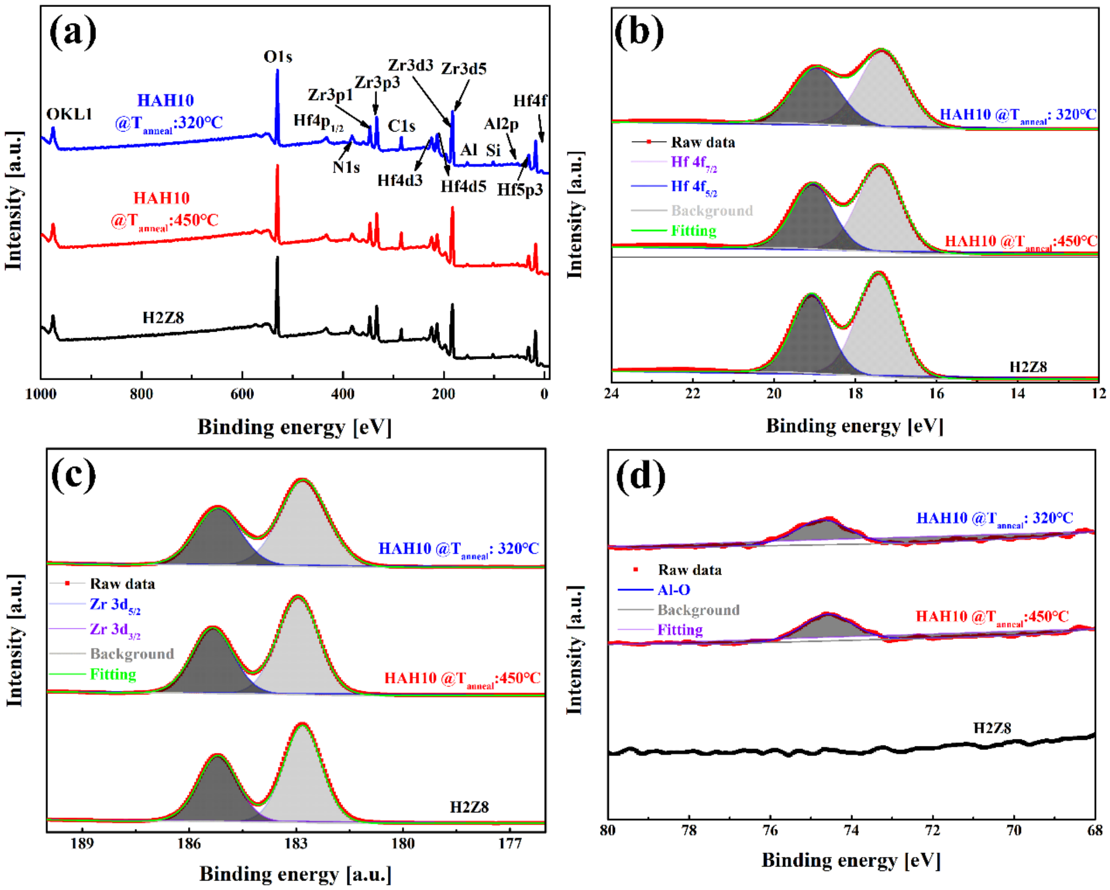

Figure S7. (a) Survey scan and high-resolution XPS results for H2Z8 and HAH10 films, (b) Hf 4f, (c) Zr 3d and (d) Al 3p.

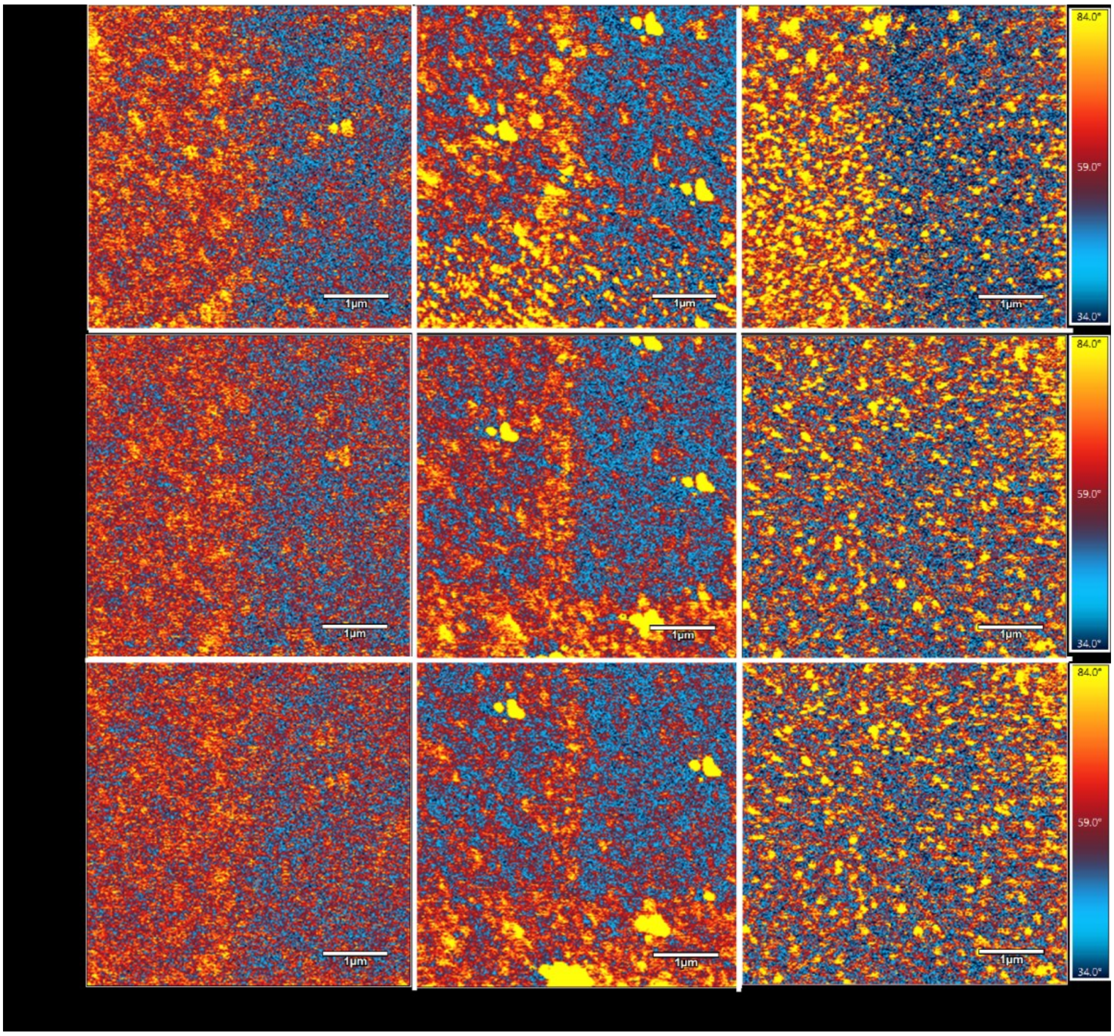

Figure S8. PFM phase contrast of reversed ferroelectric domains. (a) H2Z8, (b) HAH10 (@450 ℃), (c) HAH10 (@320 ℃). All domains above were switched by a positive tip bias of +12 V in a 2.5×2.5 μm^2 left square and a negative tip bias of -12 V in a 2.5×2.5 μm^2 square. These images were measured just after the domains were switched, then after 2 min and 5 min.

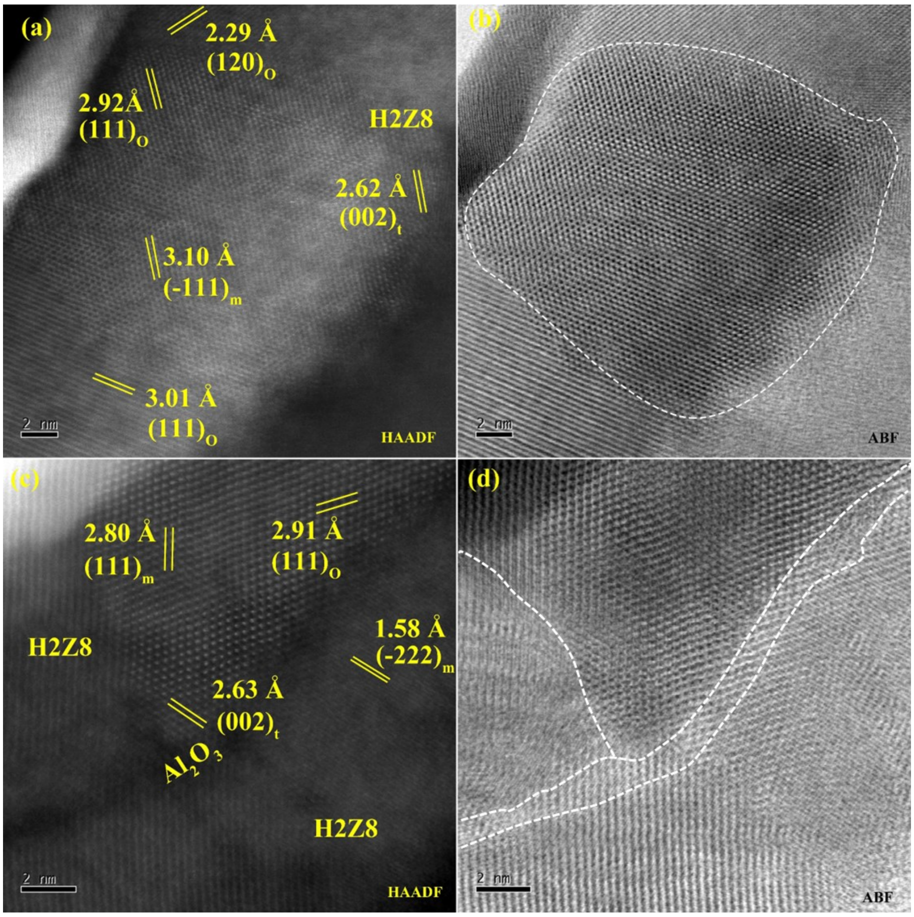

Figure S9. HRTEM images of H2Z8 (top) and HAH10 (@320℃) films (bottom) in other regions.

(a)(c) HADDF-STEM images and (b)(d) ABF-STEM images.
